# Caffeine and Parkinson’s Disease: Multiple Benefits and Emerging Mechanisms

**DOI:** 10.3389/fnins.2020.602697

**Published:** 2020-12-17

**Authors:** Xiangpeng Ren, Jiang-Fan Chen

**Affiliations:** ^1^Molecular Neuropharmacology Lab, School of Optometry and Ophthalmology, Wenzhou Medical University, Wenzhou, China; ^2^State Key Laboratory of Ophthalmology, Optometry and Visual Science, Wenzhou, China; ^3^Department of Biochemistry, Medical College, Jiaxing University, Jiaxing, China

**Keywords:** caffeine, Parkinson’s disease, α-synuclein, adenosine A_2A_ receptor, autophagy, gut microbiota

## Abstract

Parkinson’s disease (PD) is the second most common neurodegenerative disorder, characterized by dopaminergic neurodegeneration, motor impairment and non-motor symptoms. Epidemiological and experimental investigations into potential risk factors have firmly established that dietary factor caffeine, the most-widely consumed psychoactive substance, may exerts not only neuroprotective but a motor and non-motor (cognitive) benefits in PD. These multi-benefits of caffeine in PD are supported by convergence of epidemiological and animal evidence. At least six large prospective epidemiological studies have firmly established a relationship between increased caffeine consumption and decreased risk of developing PD. In addition, animal studies have also demonstrated that caffeine confers neuroprotection against dopaminergic neurodegeneration using PD models of mitochondrial toxins (MPTP, 6-OHDA, and rotenone) and expression of α-synuclein (α-Syn). While caffeine has complex pharmacological profiles, studies with genetic knockout mice have clearly revealed that caffeine’s action is largely mediated by the brain adenosine A_2A_ receptor (A_2A_R) and confer neuroprotection by modulating neuroinflammation and excitotoxicity and mitochondrial function. Interestingly, recent studies have highlighted emerging new mechanisms including caffeine modulation of α-Syn degradation with enhanced autophagy and caffeine modulation of gut microbiota and gut-brain axis in PD models. Importantly, since the first clinical trial in 2003, United States FDA has finally approved clinical use of the A_2A_R antagonist istradefylline for the treatment of PD with OFF-time in Sept. 2019. To realize therapeutic potential of caffeine in PD, genetic study of caffeine and risk genes in human population may identify useful pharmacogenetic markers for predicting individual responses to caffeine in PD clinical trials and thus offer a unique opportunity for “personalized medicine” in PD.

## Introduction

Parkinson’s disease (PD) is the second most common neurodegenerative disorder characterized by clinical presentation of motor impairment and non-motor symptoms. The hallmarks of PD pathology are selective degeneration of dopaminergic neurons in the midbrain and by the prominent α-Syn-containing proteinaceous inclusions, Lewy body (Dauer and Przedborski, 2003; Kalia and Kalia, 2015; Postuma and Berg, 2016; Wong and Krainc, 2017). PD affect 1% of the world population age 65 and older, but with the aging population and increases in life expectancy in modern society, the number of PD cases is projected to double by 2030 (Dorsey et al., 2007; Lees et al., 2009). Dopamine replacement, such as L-dopa, is the mainstay treatment to control motor symptoms (Rascol et al., 2002; Lang and Obeso, 2004; Goetz et al., 2005), but chronic L-dopa treatment is associated with loss of its efficacy and the onset of debilitating motor complications (dyskinesia, wearing-off and ON-OFF) (Ahlskog and Muenter, 2001). Furthermore, with the discovery of α-Syn as the main pathogenesis gene and the main component of Lewy body (Lashuel et al., 2013; Kalia and Kalia, 2015; Wong and Krainc, 2017), wide-spread distribution of α-Syn aggregates in the brain provide the pathological basis for non-motor symptoms of PD (e.g., cognitive dysfunction, fatigue, balance impairment, sleep disturbance, autonomic dysfunction) which are increasingly recognized as a key component of the disease (Chaudhuri et al., 2006; Postuma and Berg, 2016). Importantly, despite of the extensive studies, there are no successful treatments currently available that can slow or halt this chronic neurodegeneration (Shoulson, 1998; Mattson, 2004; Olanow, 2004; Lopez-Diego and Weiner, 2008; Mestre et al., 2009).

In the absence of an effective disease modifying treatment to slow down or hold PD course, epidemiological and experimental investigations into potential risk factors (such as dietary factor caffeine) that may modify PD pathology and confer therapeutic benefits become compelling. Caffeine is probably the most widely psychoactive substance and regularly consumed by >50% of the world’s adult population, largely for its psychostimulant (and cognitive enhancement) effect, reducing fatigue and enhancing performance. The regular human consumption of caffeine was previously estimated to be 70–350 mg/person/day or 5 to 8 mg/kg/day (equivalent to three couples of coffee) which is absorbed by the small intestine within 45 min of ingestion and produces peak plasma concentration of 0.25 to 2 mg/l (or approximately 1 to 10 μM) within 1–2 h, distributes throughout all bodily tissues and produces overall psychostimulant effects, plasma caffeine levels are usually in the range of 2–10 mg/L in coffee drinkers (Nehlig et al., 1992; Fredholm et al., 1999b). Caffeine-containing drinks, such as coffee, tea, and cola, are very popular; as of 2014, 85% of American adults consumed some form of caffeine daily, consuming 164 mg on average (Mitchell et al., 2014). Caffeine intake doses determined not to be associated with adverse effects by Health Canada (comparators: 400 mg/day for adults [10 g for lethality], 300 mg/day for pregnant women, and 2.5 mg/kg/day for children and adolescents), 2.5 mg caffeine/kg body weight/day remains an appropriate recommendation (Wikoff et al., 2017). In healthy adults, caffeine’s half-life is between 3 and 7 h, caffeine is metabolized in the liver by the cytochrome P450 oxidase enzyme system, in particular, by the CYP1A2 isozyme, into three dimethylxanthines, including paraxanthine, theobromine, and theophylline, each of which has its own effects on the human body. Caffeine was found to present in all rat tissues after administration for 10 days and accumulated for 25 days. The caffeine level was high in brain, liver and kidney and widely distributed and accumulated in these organs (Che et al., 2012). Because caffeine is both water- and lipid-soluble, it readily crosses the blood brain barrier. Once in the brain, caffeine may act at multiple molecular targets to produce complex pharmacological actions, ranging from adenosine receptor antagonism, to phosphodiesterase inhibition, to GABA receptor blockade and calcium release (Fredholm et al., 1999a). However, genetic knockout studies have demonstrated that caffeine’s primary action in the brain is as an antagonist of adenosine receptors (mainly A_2A_R) (Chen et al., 2013).

In this review, we first summarized the neuroprotective, motor and cognitive benefits of caffeine in PD patients and PD models. We then described the potential mechanisms underlying caffeine’s protective effects, including modulation of neuroinflammation and of newly emerging mechanisms associated with autophagy and gut microbiota. Lastly, we discussed possible genetic polymorphisms of caffeine-associated genes in influencing caffeine drug responses and its clinical implications.

## Multiple Benefits of Caffeine in PD Treatment

### Potential Neuroprotective Effects of Caffeine in PD Patients and PD Models

The first evidence for the potential neuroprotective effect of caffeine came from the Honolulu Heart Program, a large prospective study of 8004 Japanese-American men over a 30 years follow-up study. The study revealed that daily consumption of 784 mg/kg or more of coffee during the mid-life reduce the risk for developing PD at age of 65-year old by 5-folds compared to non-coffee drinkers after age- and smoking-adjustment (Ross et al., 2000). This inverse relationship between consumption of caffeine and the risk of developing PD was further supported by the Health Professionals’ Follow-Up Study and the Nurses’ Health Study – involving 47,351 men and 88,565 women, and subsequently also by at least three additional large prospective studies, including Finnish Mobile Clinic Health Examination Survey (Saaksjarvi et al., 2008) and the NeuroGenetics Research Consortium (Powers et al., 2008) and Danish case-control study involving idiopathic PD (Kyrozis et al., 2013). A meta-analysis of 13 study involving total 901,764 participants for coffee intake found a non-linear relationship was found between coffee intake and PD risk, with maximum protection effect at approximately 3 cups/day (Qi and Li, 2014). Systemic analysis of 120 observation studies have firmly established that regular human consumption of caffeine is associated with reduced risk for PD (Ross et al., 2000; Ascherio et al., 2001, 2003; Saaksjarvi et al., 2008; Grosso et al., 2017) and does not impose significant adverse effects on the cardiovascular system, bone status, or the incidence of cancer (Fredholm et al., 1999b; Winkelmayer et al., 2005; Higdon and Frei, 2006; van Dam et al., 2006; Cadden et al., 2007; Daly, 2007). Interestingly, this inverse relationship of coffee consumption and risker for developing PD is largely attributed to caffeine since that the consumption of caffeinated (but not decaffeinated) coffee is associated with the reduced risk of developing PD in the Health Professional Follow-up Study (Ascherio et al., 2001). Notably, this inverse relationship is strong and consistent in men in the Health Professional Follow-up Study (Grosso et al., 2017) and in postmenopausal women who never used hormone replacement therapy in the Cancer Prevention Study II Nutrition Cohort (CPS II-Nutrition) (Palacios et al., 2012), but uncertain in women and postmenopausal women ever users of hormone replacement therapy. Thus, caffeine and estrogen interaction modify the risk of developing PD. More recently, the Harvard Biomarkers Study (HBS) conducted a cross-sectional, case-control study in 566 subjects consisting of idiopathic PD patients and healthy controls, which highlighted the robustness of lower caffeine intake and plasma urate levels as factors inversely associated with idiopathic PD (Bakshi et al., 2020).

Furthermore, the neuroprotective effect of caffeine from epidemiological investigation is further supported by mounting evidence from animal studies demonstrating that caffeine confers neuroprotection against dopaminergic neurodegeneration in neurotoxin PD models using mitochondrial toxins (MPTP, 6-OHDA and rotenone) (Chen et al., 2001; Ikeda et al., 2002; Xu et al., 2002; Aguiar et al., 2006) and α-Syn transmission mouse model through intracerebral injection of α-Syn fibers (Luan et al., 2018). For example, acute or chronic treatment with caffeine attenuates MPTP-induced dopaminergic neurotoxicity and neurodegeneration (Chen et al., 2001; Xu et al., 2002). It is interesting to note that in a chronic MPTP infusion model of PD caffeine can still confer protection against dopamine neurodegeneration even when caffeine was administered after the onset of the neurodegenerative process (i.e., 14 days after MPTP infusion) (Sonsalla et al., 2012). Furthermore, our recent study demonstrated that caffeine can protect against A53T mutant α-Syn induced pathological alterations in intact animals using the α-Syn fibril model of PD, an effect associated with the enhanced activity of autophagy (specifically macroautophagy and CMA) (Luan et al., 2018) (see below for the more discussion).

Importantly, similar to caffeine, pharmacological blockade or genetic deletion of adenosine A_2A_ receptor (A_2A_R, the main pharmacological target of caffeine in the brain) also protected against dopaminergic neurodegeneration in animal models of PD (Chen et al., 2001; Ikeda et al., 2002; Kachroo and Schwarzschild, 2012), suggesting that the protective effects of caffeine are likely due to its action on the A_2A_R. Moreover, recent studies also show that A_2A_R modulated α-Syn aggregation and toxicity in SH-SY5Y cells (Ferreira et al., 2017a), and A_2A_R blockade rescued synaptic and cognitive deficits in α-Syn transgenic mice model of PD (Ferreira et al., 2017b). These animal studies provide a neurobiological basis for the inverse relationship between caffeine consumption and the reduced risk of developing PD, and support the clinical potential for caffeine and A_2A_R antagonists as a disease-modifying drug target for PD.

Two important caveats need to be taken into the consideration. First, caffeine exerts synergistic neuroprotection with eicosanoyl-5-hydroxytryptamide (EHT) in animal models of PD, co-administration of these two compounds of coffee orally have synergistic effects in protecting the brain against α-Syn mediated toxicity through maintenance of protein phosphatase 2A in an active state (Yan et al., 2018). Second, in methamphetamine-induced neurotoxicity both *in vitro* and *in vivo*, caffeine has been shown to increase toxicity of methamphetamine in SH-SY5Y neuroblastoma cell line through inhibition of autophagy (Pitaksalee et al., 2015) and potentiate 3,4-methylenedioxymethamphetamine (MDMA) -induced dopamine neuron degeneration in substantia nigra pars compacta, possibly involving an increase in dopamine release and excess ROS generation (Sinchai et al., 2011; Frau et al., 2016). Therefore, caffeine has been consistently shown to exert a neuroprotective effect in multiple neurotoxin (including MPTP, 6-OHDA, rotenone) and alpha-synuclein models of PD, but has been reported to exacerbate methamphetamine-induced neurotoxicity. Additional studies are needed to clarify these neurotoxicity context-dependent effects of caffeine.

### Motor Benefit of Caffeine in PD Patients and PD Models

The symptomatic effect of caffeine in PD was first tested in 1970s (Shoulson and Chase, 1975), but has been revisited by several clinical studies recently. The motor benefit of caffeine were documented in a pilot open-label, 6-week dose-escalation study (Altman et al., 2011) and a 6-week randomized controlled trial of caffeine (200–400 mg daily) involving 61 PD patients (Postuma et al., 2012). These clinical studies suggest that caffeine improved objective motor deficits in PD with the reduced total Unified PD Rating Scale score and the objective motor component. Furthermore, coffee consumption (>336 mg/day) is associated with the reduced hazard ratio for the development of dyskinesia compared with subjects who consumed <112 mg/day in the Comparison of the Agonist Pramipexole with Levodopa on Motor Complications of Parkinson’s Disease (CALM-PD) and CALM Cohort extension studies (Wills et al., 2013). Based on these positive findings, caffeine was recently investigated for motor and disease-modification involving 121 PD patients PD in a phase 3, 5-years (planned), two-arm, double-blind RCT, with a primary outcome focused on motor symptoms and disease-modification as a secondary outcome^1^. Unfortunately, with the primary outcome analysis after 6 months demonstrating no significant symptomatic benefit of caffeine (200 mg twice daily) (Postuma et al., 2017), the study was terminated earlier than the planned.

Based on the concentrated expression of A_2A_R in the striatum and the A2AR is the key molecular target of caffeine, caffeine (and A_2A_R antagonists) has been proposed 20 years ago to improve motor activity in PD (Garcao et al., 2013; Morato et al., 2017). Indeed, preclinical studies using rodent and non-human primate models of PD demonstrate motor benefits caffeine and A_2A_R antagonists in PD (Richardson et al., 1997; Chen, 2003; Schwarzschild et al., 2006; Jenner et al., 2009), leading to clinical pursuit of A_2A_R antagonists as a leading non-dopaminergic treatment for motor deficits in PD. Since 2001, more than 25 clinical trials were conducted to evaluate the safety and clinical efficacy of A_2A_R antagonists in PD patients; among these, eight double blind, placebo controlled, phase IIb and III trials of istradefylline (KW-6002, > 4000 PD patients), one phase IIb trial with preladenant (SCH 420814, 253 PD patients), one phase IIb trial with tozadenant (337 PD patients), all reported motor benefits in advanced PD patients as an add-on therapy with L-DOPA (Hauser et al., 2011). The culmination of the two decade-long clinical studies of the effects of istradefylline in more than 4,000 PD finally led to the U.S. Food and Drug Administration (FDA) approval of the A_2A_R antagonist Nourianz^®^ (istradefylline) developed by Kyowa Hakko-Kirin Inc., Japan, as an add-on treatment to levodopa in Parkinson’s disease (PD) with “OFF” episodes in August 2019. Istradefylline is the first non-dopaminergic drug approved by United States FDA for PD in the last two decades and this approval paves the way to foster novel therapeutic opportunities for A_2A_R antagonists including caffeine for neuroprotection or reversal of mood and cognitive deficits in PD.

### Caffeine and Cognitive Improvement in PD

Parkinson’s disease is primarily characterized by cardinal motor symptoms but cognitive changes also occur both in the early and later stages of the disease process. In fact, ∼30% of PD patients have dementia and an additional 25% of non-demented PD patients developed mild cognitive impairments that are characterized by frontostriatal cognitive deficits such as alterations in executive function, attention, working and episodic memory (Majbour and El-Agnaf, 2017). Convergent evidence from human and animal studies supports the existence of DA-dependent cognitive deficits in PD (Lewis et al., 2003). The cognitive symptoms seen early in PD include the decreased executive function (e.g., planning and decision making), working memory deficit and impaired procedural learning, leading to cognitive inflexibility that are largely attributed to the frontostriatal dysfunction (Knowlton et al., 1996; Kehagia et al., 2010). Early cognitive deficits are extremely troubling to patients and reduce their quality of life. Currently there is no effective treatment for mild cognitive impairments in PD. In this regard, it is important to note that at least six longitudinal studies support an inverse relationship between caffeine consumption and decreased memory impairments associated with aging as well as a reduced risk of developing dementia and Alzheimer’s disease, including the Maastricht Aging Study (Hameleers et al., 2000; van Boxtel et al., 2003), the Canadian Study of Health and Aging (CSHA) (Lindsay et al., 2002), the FINE study (van Gelder et al., 2007), the French Three Cities study (Ritchie et al., 2007), the Cardiovascular risk factors, Aging and Dementia (CAIDE) study (Eskelinen et al., 2009), the Honolulu-Asia Aging Study (Gelber et al., 2011). Furthermore, in a cross-sectional study involving 196 early-stage, treatment-naïve PD patients, coffee drinking was significantly associated with a reduced severity of the mood/cognition domain of NMSS in patients with PD (*p* = 0.003) (Cho et al., 2018). These epidemiological findings raise the possibility of caffeine as therapeutic treatment for cognitive impairments in PD.

Indeed, preclinical studies with A_2A_R antagonist effect on cognition in normal and MPTP-treated non-human primates (NHP) provide the experimental evidence that A_2A_R antagonists including caffeine can improve cognitive impairments in PD models (Chen et al., 2013; Chen, 2014). Recent preclinical studies in rodents and non-human primates demonstrated that A_2A_R antagonists not only enhance working memory (Zhou et al., 2009), reversal learning (Wei et al., 2011), set-shifting (Mingote et al., 2008), goal-directed behavior (Li et al., 2016), and Pavlovian conditioning (Wei et al., 2014) in normal animals, but also reverse working memory impairments in animal models of PD (Ko et al., 2016) and Huntington’s disease (Li et al., 2015), traumatic brain injury (Ning et al., 2013, 2015, 2019) as well as Alzheimer’s disease (AD) (Dall’Igna et al., 2007; Cunha and Agostinho, 2010; Laurent et al., 2014; Faivre et al., 2018). Furthermore, we recently demonstrated a pro-cognitive effect in normal as well as MPTP-treated Cynomolgus monkeys (Li et al., 2018b). The demonstrated treatment paradigm of the A_2A_R antagonist KW6002 for spatial working memory enhancement in non-human primate model of PD provide required preclinical data to facilitate the design of clinical trial of A_2A_R antagonists including caffeine for cognitive benefit in PD patients (Li et al., 2018b). Notably, recent clinical trials of A_2A_R antagonists for motor benefits in PD did not evaluate their possible effects on cognitive impairment in PD patients (Chase et al., 2003; Aarsland et al., 2010). With the approval of istradefylline, it will now be possible to evaluate the ability of A_2A_R antagonists to reverse cognitive deficits in PD patients in clinical phase IV trials.

## Mechanisms of Neuroprotection by Caffeine in PD

Multiple mechanisms have been proposed to account for the neuroprotective effects of caffeine, including modulation of glutamatergic excitotoxicity and neuroinflammation via adenosine receptors (Chen et al., 2013). Furthermore, recent investigation into the autophagy and gut microbiota in PD pathogenesis raise the exciting possibilities that caffeine may modify autophagy (through metabolism-related action of caffeine) and gut microbiome (with caffeine direct action on gut microbiota) to influence PD development.

### Caffeine Modulates Neuroinflammation in PD

Neuroinflammation is critically involved in the pathogenesis of PD. Increasing evidence showed that neuroinflammation response regulated by reactive microglia played an important role in the neurodegeneration of DA neurons (Hirsch and Hunot, 2009; Tansey and Goldberg, 2010; Hirsch et al., 2012). α-Syn, in extracellular aggregated form, can bind to Toll-like receptor 2 (TLR2), CD11b receptors and integrin β1 subunit on microglia to trigger massive microglial activation and neuroinflammation, contributing to consequent neuronal death (Lee et al., 2010; Tansey and Goldberg, 2010; Fellner et al., 2013; Yasuda et al., 2013; Sacino et al., 2014). The involvement of neuroinflammation in PD was further suggested by the observation of the increased number of reactive microglial cells and an upregulation of major histocompatibility complex class II (MHC-II) in PD patients (McGeer et al., 1988). Caffeine can exert an anti-neuroinflammatory effect under various pathological conditions. Caffeine administration (daily intraperitoneal injection) reduces lipopolysaccharide (LPS)-induced microglia activation in three regions of the hippocampus, in a dose-dependent manner (Brothers et al., 2010) and abrogate LPS-induced neuroinflammation, and synaptic dysfunction in adult mouse brains (Badshah et al., 2019). As a critical neuroprotective factor in PD, caffeine may control microglia-mediated neuroinflammatory response associated PD (Madeira et al., 2017). Indeed, daily intraperitoneal administration of caffeine attenuates microglia reactivity and prevents blood-brain barrier (BBB) dysregulation in the MPTP mouse model, leading to decreased dopaminergic neuronal loss (Xu et al., 2002; Chen et al., 2008). Furthermore, even when introduced in the later phases of the neurodegenerative process, caffeine is also able to attenuate the inflammatory process and microglial cell expression of CD68 (a marker of reactive microglia), which suggests its ability to arrest or delay neuroinflammation and neurodegeneration (Chen et al., 2008). Consistent with this view, by using an α-Syn fibril model of PD, we recently found that chronic caffeine treatment attenuated α-Syn-induced microglial activation and astrogliosis in the striatum in mice (Luan et al., 2018). In addition, caffeine has been shown to protect dopaminergic neurons by activation of the anti-oxidative signaling pathways, such as the nuclear factor erythroid 2-related factor 2 (Nrf2)-Keap1 and peroxisome proliferator-activated receptor gamma coactivator 1-alpha (PGC-1 alpha) (Zhou et al., 2019).

The mechanisms underlying anti-neuroinflammation by caffeine may involve the antagonism of the A_2A_R, the major molecular target (Chen et al., 2001; Kalda et al., 2006). A_2A_Rs affords neuroprotection through the control of microglia reactivity and neuroinflammation. Notably, pharmacological blockade or genetic deletion of A_2A_R produces similar anti-neuroinflammatory and neuroprotective effects as with caffeine in several experimental models of PD (Chen et al., 2001; Ikeda et al., 2002; Kalda et al., 2006; Hu et al., 2016; Luan et al., 2018). For example, enhanced reactive astrogliosis and NF-κB p65 activation around the injection site in hippocampus in an α-Syn transmission mouse model of PD, and these α-Syn-triggered neuroinflammatory responses were largely prevented in A_2A_R KO mice (Hu et al., 2016). In the well-established α-Syn fibril model of PD, chronic caffeine treatment largely reverted the α-Syn-induced microglial activation and astrogliosis in the striatum in mice (Brambilla et al., 2003; Boison et al., 2010; Luan et al., 2018). Moreover, in A_2A_R antagonists also control neuroinflammation (through p38), of synaptopathy (Canas et al., 2009) and β-amyloid processing (Cao et al., 2009) in AD models. Thus, caffeine may exert anti-neuroinflammation and neuroprotection effect in PD by targeting the A_2A_Rs.

### Caffeine May Modulate PD Pathology by Regulating Autophagy Activity

Autophagy is a high conserved defense and protective mechanism in eukaryotic cell to achieve degradation of abnormal proteins and damaged organelles by three types of autophagy processes: microautophagy, macroautophagy and chaperone-mediated autophagy (CMA). PD is essentially a protein misfolding disease or conformational disease; characterized by abnormal accumulation and aggregation of misfolded α-Syn (Kalia and Kalia, 2015). Increasing evidence demonstrates that aberrant regulation of autophagy contributes to the aggregation of α-Syn and α-Syn-induced neurodegeneration in PD (Ebrahimi-Fakhari et al., 2012; Poehler et al., 2014; Xilouri et al., 2016; Hou et al., 2020; Senkevich and Gan-Or, 2020). First, several studies found that alteration of autophagy-lysosome pathway activity, including both CMA and macroautophagy, existed in brains of postmortem PD patients and experimental models of PD (Alvarez-Erviti et al., 2010; Cerri and Blandini, 2019; Lamonaca and Volta, 2020; Qin et al., 2020; Wan et al., 2020), revealing the direct correlation between autophagy and pathological process of PD. Second, abnormally aggregated α-Syn inclusions are mainly degraded by autophagy (Webb et al., 2003) and defects or deficiency of autophagy can lead to accumulation of intracellular misfolded amyloid α-Syn aggregates, thus causally linking autophagy to PD pathological process (Xilouri et al., 2016; Guo et al., 2020). Third, α-Syn overexpression can impede autophagy by reducing autophagosome formation in human Neuroblastoma SH-SY5Y (Nascimento et al., 2020), and contribute to many different pathologies seen in PD (Winslow and Rubinsztein, 2011). Notably, the pathogenic A53T and A30P α-Syn mutants selectively bound to lysosome-associated membrane protein type 2A (LAMP2A) with high affinity, blocked CMA activity at the LAMP2A level, ultimately resulting in complete damage to this pathway (Cuervo et al., 2004). Collectively, autophagy plays an important role in the pathogenesis of PD, thus targeting autophagy may represent a promising strategy for treatment of PD (Rivero-Rios et al., 2016; Moors et al., 2017; Lu et al., 2020).

Both caffeine and A_2A_R signaling can regulate autophagy activity under different conditions in several cell types (Sinha et al., 2014; Liu et al., 2016). Caffeine can induce macroautophagy caused by a starvation response and confer a cytocidal effect on Zygosaccharomyces bailii food spoilage yeast) in combination with benzoic acid (Winter et al., 2008). Higher concentrations of caffeine (10 mM) enhance autophagic flux in various tumor cell lines (HeLa, SH-SY5Y, and PC12D cells). Caffeine can induce apoptosis by enhancement of autophagy in PC12D cells through PI3K/Akt/mTOR/p70S6 signaling pathway (Saiki et al., 2011). Using both genetic and pharmacological inhibitors of autophagy, researchers directly linked caffeine-induced autophagy with oxidative lipid metabolism both in HepG2 cells and in mice liver; and further demonstrated that autophagy was associated with caffeine-induced hepatic fat clearance in a mouse model of non-alcoholic fatty liver disease, indicating that caffeine surprisingly is a potent stimulator of hepatic autophagic flux (Ray, 2013; Sinha et al., 2014). Accordingly, caffeine-enhanced autophagic flux in hepatic stellate cells was stimulated by endoplasmic reticulum (ER) stress via the IRE1-α signaling pathway, and autophagy trigged by caffeine instigated cell apoptosis (Li et al., 2017). Acute high-caffeine exposure also significantly reduced skeletal myotube diameter by increasing autophagic flux in differentiated C2C12 mouse skeletal myoblasts cells (Bloemberg and Quadrilatero, 2016; Hughes et al., 2017). Notably, caffeine increased autophagy by promoting the calcium-dependent activation of AMP-activated protein kinase (AMPK) in mammalian skeletal muscle cells (Mathew et al., 2014), and prevent skin from oxidative stress-induced senescence through the activation of autophagy. This caffeine-induced autophagy, mainly mitophagy, was mediated by A_2A_R/SIRT3/AMPK pathway, protecting skin from oxidative stress-induced senescence both *in vitro* and *in vivo* models (Li et al., 2018a). Therefore, caffeine also has the potential in protection of skin disease. Consistent with these studies, a recent research found that caffeine directly enhanced autophagy in concentration- and time-dependent manners in primary cultured thymocytes, which was dependent on A_2A_R signaling (Liu et al., 2019). Taken together, these studies from various cell models and in different tissues and organs *in vivo* demonstrate that caffeine enhances autophagy which is related to its therapeutic effects on diverse human diseases, including tumors, aging, liver fibrosis, and skin disease.

Using an α-Syn fibril model of PD, we recently have provided the first evidence that caffeine can attenuate abnormal α-Syn aggregation and neurotoxicity by re-establishing autophagy activity in animal models of PD (Luan et al., 2018). Specifically, chronic caffeine treatment did not affect autophagy processes in the normal mice striatum, but did selectively reverse α-Syn-induced defects in macroautophagy and CMA (Luan et al., 2018). Thus, caffeine may represent a novel pharmacological therapy for PD by targeting autophagy pathway. This study collaborates with the previous study showing that caffeine-induced autophagy protected against human prion protein (PrP) peptide (106–126)-triggered apoptosis in a SH-SY5Y neuroblastoma cell line. Therefore, autophagy enhanced by caffeine may be a valid therapeutic strategy for neurodegenerative diseases such as PD and prion (Moon et al., 2014; Corti et al., 2020).

### Caffeine May Influence PD Pathology by Modulating Gut Microbiota

The gut microbiota in the human gastrointestinal (GI) tract is estimated to contain 10 times more microbial cells than human cells, and approximately 100–200-times more protein coding genes than the human genome (Qin et al., 2010). The gut microbiota critically influence various aspects of human biology, including the absorption and metabolism of nutrients, vitamins, medications, and toxic compounds; the development and differentiation of the intestinal epithelium and immune system, the maintenance of tissue homeostasis, and the prevention of pathogens invasion (Sommer and Backhed, 2013). The gut microbiota also plays an important role in gut-brain communication, and the neuroimmune system to maintain brain homeostasis, thus influencing brain function and behavior (Carabotti et al., 2015). In healthy subjects, the intestinal microbiota is generally stable over time, but compositional changes might occur following antibiotic usage or dietary modifications (Lozupone et al., 2012).

Mounting evidence suggest that the intestinal microbiota may be the triggering factor of PD pathology. Specifically, the gut microbiota encoded proteins and their metabolites can initiate accumulation of misfolding of α-Syn in the enteric nervous system through molecular mimicry and intestinal mucosal immunoinflammatory mechanisms, which thereafter could act in a prion-like fashion and spread along the gut-brain axis via vagus nerve, eventually leading to the development of PD pathology (Friedland, 2015; Klingelhoefer and Reichmann, 2015; Pellegrini et al., 2018; Ho et al., 2019; Miraglia and Colla, 2019; Cirstea et al., 2020; Zheng et al., 2020). Multiple lines of preclinical and clinical evidences support the role of gut microbiota dysfunction in various aspects of PD pathogenesis: (i) according to the widely accepted Braak Staging hypothesis about the pathogenesis of PD, α-Syn pathology may begin in the intestine and spread to the brain through the vagus nerve. This hypothesis has been supported by two lines of key clinical findings. (a) Idiopathic constipation is one of the strongest risk-factors for PD since up to 80% of PD patients develop gastrointestinal dysfunction, in particular constipation, in the early stages of PD, preceding the onset of motor symptoms by years (Schapira et al., 2017). (b) Full truncal vagotomy is associated with a decreased risk for subsequent PD, suggesting that the vagal nerve may be critically involved in the pathogenesis of PD (Svensson et al., 2015). (ii) The gut microbiota of patients with PD is altered depending on clinical motor phenotype and related to progress of PD; thus the gut microbiome may be a potential PD biomarker (Scheperjans et al., 2015a; Hopfner et al., 2017; Gerhardt and Mohajeri, 2018; Qian et al., 2018; Nuzum et al., 2020; Pietrucci et al., 2020; Ren et al., 2020). (iii) Gut microbiota is necessary to promote α-Syn pathology, neuroinflammation, neurodegeneration, and characteristic motor features in validated mice model of PD, and specific microbial metabolites are sufficient to promote PD symptoms, suggesting a casual and functional role in PD pathogenesis (Sampson et al., 2016; Sun et al., 2018; Klann et al., 2020; Koutzoumis et al., 2020). (iv) The human gut microbiota metabolizes the PD medication Levodopa (L-dopa), potentially reducing drug availability and causing side effects (Maini Rekdal et al., 2019). Collectively, these findings support that gut microbiota is critical contributor to PD pathogenesis and may represent a promising therapeutic target for the treatment of PD (Lubomski et al., 2019).

As the most important environmental and dietary factor of PD, how does long-term drinking caffeine affect the diversity of gut microbiota? Moreover, how do the human intestinal microorganism and their encoded enzymes influence the metabolism of caffeine? Caffeine is initially absorbed in the stomach and small intestine but is further fermented in the colon by gut microbiota (Scheperjans et al., 2015b). Recently, caffeine consumption is reportedly related to the colonic mucosa-associated gut microbiota (Gurwara et al., 2019); long-term coffee intake is associated with fecal microbial composition in humans, and regular consumption of coffee appears to be associated with changes in some intestinal microbiota groups in which caffeine, as the main dietary factor influencing PD development, may play a role (Gonzalez et al., 2020). Intestinal microorganisms also play a role in the metabolism of caffeine as caffeine was degraded in the gut of *H. hampei*, and that experimental inactivation of the gut microbiota eliminates this activity, suggesting that the detoxification of caffeine in *H. hampei* is mediated by the insect’s gut microbiota (Ceja-Navarro et al., 2015). Several recent studies have shed light on the relationship between coffee consumption, caffeine and gut microbiota, as well as GI function in PD (Derkinderen et al., 2014; Scheperjans et al., 2015b). Some effects of coffee on the gastrointestinal tract promote gastro-oesophageal reflux, stimulation of gallbladder contraction and an increase of colonic motor activity (Boekema et al., 1999). Coffee consumption is also inversely associated with the prevalence of self-reported constipation (Murakami et al., 2006). Notably, coffee and caffeine regulates the composition and abundance of intestinal flora under different pathologies. For example, coffee caused an increase of anti-inflammatory Bifidobacteria and a decrease of Clostridium spp. and Escherichia coli that invade the gut mucosa in PD (Khokhlova et al., 2012). Furthermore, chronic coffee consumption attenuated the increase in Firmicutes (F)-to-Bacteroidetes (B) ratio and Clostridium Cluster XI normally associated with high-fat feeding and augmented the levels of Enterobacteria (Cowan et al., 2014). Coffee or its components caffeine can also affect the gut microbiome and short-chain fatty acids (SCFAs) profile in Tsumura Suzuki obese diabetes (TSOD) mice and thereby improve hepatic inflammation. Specifically, daily intake of coffee or its components did not repair the gut dysbiosis in TSOD mice, rather, altered the percentages of six microbial genera changed in these mice, including Blautia, Coprococcus, and Prevotella, which have been implicated in inflammation or obesity (Clarke et al., 2012; Nishitsuji et al., 2018). Other studies suggest that the diversity and structure of the gut microbiota is not sensitive to caffeine, however, when the predicted metagenome functional content of the bacterial communities was analyzed, the caffeine treatments did induce a dramatic decrease of the aromatic amino acid decarboxylase gene (Scorza et al., 2019).

Consistent with these findings, our preliminary meta-genome analysis of the gut microbiota of A53T-α-Syn transgenic mice model of PD by chronic intervention of caffeine indicated that chronic caffeine treatment for one month had no significant effect on gastrointestinal function, but apparently normalized the structure and imbalance of the gut microbiota of PD model mice (unpublished data). Additional studies are warranted to study the interaction between caffeine and intestinal flora in the body, and determine whether the beneficial effects of coffee consumption on PD are mediated through the modulation of the microbiota-gut-brain axis.

## Genetic Studies on the Interaction Between Caffeine and PD Risk Genes

Epidemiological investigation coupled with genetic analyses of the genetic and environmental interaction in development of PD has provided several important insights into the interaction between caffeine and several genes associated with PD pathogenesis such as NMDA-glutamate-receptor subunit, LRRK2 and estrogen in PD: (i) A recent genome-wide association and interaction study (GWAIS) uncover a complex interplay between genetic (GRIN2A, encoding an NMDA-glutamate-receptor subunit) and environmental factors (caffeine consumption) in etiology of PD, as a PD genetic modifier in inverse association with caffeine intake (Hamza et al., 2011). (ii) A study with patient-control study Swedish population has revealed an association of a single nucleotide polymorphism, GRIN2A_rs4998386, and its interaction with caffeine intake with PD (Yamada-Fowler et al., 2014). Thus, the interaction between caffeine and glutamate receptor genotypes may contribute to the protective effects of coffee drinking/caffeine intake in PD. (iii) Furthermore, a recent case control study of 812 subjects consisting of PD and healthy controls showed that caffeine intake would significantly reduce the risk of PD by 15-folds in those carrying PD gene risk variant (LRRK2 R1628P) (Kumar et al., 2015). Metabolomic analyses of the LRRK2 Cohort Consortium (LCC) samples identified caffeine, its demethylation metabolites, and trigonelline as prominent markers of resistance to PD linked to pathogenic LRRK2 mutations, more so than to idiopathic PD (Crotty et al., 2020). Exploratory analysis on potential interactions of smoking and caffeine intake with 10 genome-wide association studies of SNPs (at or near the SNCA, MAPT, LRRK2, and HLA loci) further reveal that a combined smoking and caffeine intake exposure is associated with a significant interaction with rs2896905 at SLC2A13, near LRRK2 (Gao et al., 2012). In addition, the Parkinson’s Epidemiology and Genetics Association Studies in US (PEGASUS) consortium (involving 3000 subjects of five independent well-characterized patient-control series) uncovered an association between an *Adora2a* variant (rs7165183 and rs5996696) and a reduced risk of PD (Popat et al., 2011), with the strongest coffee-PD association among those with homozygous carriers of the *CYP1A2* polymorphisms and slow metabolizers of caffeine. These genetic studies support the protective effect of caffeine intake on PD through the interaction between caffeine and GRIN2A, LRRK2, and A_2A_R. These findings raise the exciting possibility of selecting patient subpopulations by these genetic polymorphisms of the GRIN2A, LRRK2, A_2A_R, and CYP1A1 genes.

## Author Contributions

Both authors wrote and revised the manuscript. J-FC carried out the overall responsibility.

## Conflict of Interest

The authors declare that the research was conducted in the absence of any commercial or financial relationships that could be construed as a potential conflict of interest.

## Footnotes

1 https://clinicaltrials.gov/ct2/show/NCT01738178
